# Accurate and efficient numerical solutions for elliptic obstacle problems

**DOI:** 10.1186/s13660-017-1309-z

**Published:** 2017-02-03

**Authors:** Philku Lee, Tai Wan Kim, Seongjai Kim

**Affiliations:** 1 Department of Mathematics, Sogang University, Ricci Building R1416, 35 Baekbeom-ro, Mapo-gu, Seoul, 04107 South Korea; 2 Centennial Christian School International, 20 Shin Heung Ro 26-Gil, Yongsan Gu, Seoul, 140-833 South Korea; 3 Mississippi State University, Mississippi State, MS 39762-5921 USA

**Keywords:** elliptic obstacle problem, successive over-relaxation (SOR) method, gradient-weighting method, obstacle relaxation, subgrid finite difference (FD)

## Abstract

Elliptic obstacle problems are formulated to find either superharmonic solutions or minimal surfaces that lie on or over the obstacles, by incorporating inequality constraints. In order to solve such problems effectively using finite difference (FD) methods, the article investigates simple iterative algorithms based on the successive over-relaxation (SOR) method. It introduces subgrid FD methods to reduce the accuracy deterioration occurring near the free boundary when the mesh grid does not match with the free boundary. For nonlinear obstacle problems, a method of gradient-weighting is introduced to solve the problem more conveniently and efficiently. The iterative algorithm is analyzed for convergence for both linear and nonlinear obstacle problems. An effective strategy is also suggested to find the optimal relaxation parameter. It has been numerically verified that the resulting obstacle SOR iteration with the optimal parameter converges about one order faster than state-of-the-art methods and the subgrid FD methods reduce numerical errors by one order of magnitude, for most cases. Various numerical examples are given to verify the claim.

## Introduction

Variational inequalities have been extensively studied as one of key issues in calculus of variations and in the applied sciences. The basic prototype of such inequalities is represented by the so-called obstacle problem, in which a minimization problem is often solved. The obstacle problem is, for example, to find the equilibrium position *u* of an elastic membrane whose boundary is held fixed, with an added constraint that the membrane lies above a given obstacle *φ* in the interior of the domain $\varOmega \subset \mathbb{R}^{d}$:

1.1 $$ \min_{u} \int_{\varOmega } \sqrt{1+|\nabla u|^{2}}\, d\mathbf{x}, \quad \text{s.t. } u\ge\varphi\text{ in }\varOmega , u=f \text{ on }\varGamma , $$

where $\varGamma =\partial \varOmega $ denotes the boundary of *Ω* and *f* is the fixed value of *u* on the boundary. The problem is deeply related to the study of minimal surfaces and the capacity of a set in potential theory as well. Other classical applications of the obstacle problem include the study of fluid filtration in porous media, constrained heating, elasto-plasticity, optimal control, financial mathematics, and surface reconstruction [1–7].

The problem in (1.1) can be linearized in the case of small perturbations by expanding the energy functional in terms of its Taylor series and taking the first term, in which case the energy to be minimized is the standard Dirichlet energy

1.2 $$ \min_{u} \int_{\varOmega } |\nabla u|^{2}\, d\mathbf{x}, \quad\text{s.t. } u \ge\varphi\text{ in }\varOmega , u=f \text{ on }\varGamma . $$

A variational argument [2] shows that, away from the contact set $\{\mathbf{x}|u(\mathbf{x})=\varphi(\mathbf{x})\}$, the solution to the obstacle problem (1.2) is harmonic. A similar argument (which restricts itself to variations that are positive) shows that the solution is superharmonic on the contact set. Thus both arguments imply that the solution is a superharmonic function. As a matter of fact, it follows from an application of the maximum principle that the solution to the obstacle problem (1.2) is the least superharmonic function in the set of admissible functions. The Euler-Lagrange equation for (1.2) reads

1.3 $$ \textstyle\begin{array}{l@{\quad}l} \left . \textstyle\begin{array}{l} -\Delta u\ge0, \\ u\ge\varphi,\\ (-\Delta u)\cdot(u-\varphi)=0, \end{array}\displaystyle \right \} & \text{in }\varOmega , \\ u=f, & \text{on }\varGamma . \end{array} $$

In modern computational mathematics and engineering, the obstacle problems are not extremely difficult to solve numerically any more, as shown in numerous publications; see [8–14], for example. However, most of those known methods are either computationally expensive or yet to be improved for higher accuracy and efficiency of the numerical solution. In this article, we consider accuracy-efficiency issues and their remedies for the numerical solution of elliptic obstacle problems. This article makes the following contributions.

- *Accuracy improvement through subgrid finite differencing of the free boundary*: It can be verified either numerically or theoretically that the numerical solution easily involve a large error near the free boundary (the edges of obstacles), particularly when the grid mesh does not match with the obstacle edges. We suggest a post-processing algorithm which can reduce the error (by about a digit) by detecting accurate free boundary in subgrid level and introducing nonuniform *finite difference* (FD) method. The main goal of the subgrid FD algorithm is to produce a numerical solution of a higher accuracy $u_{h}$, which guarantees $u_{h}(\mathbf{x})\ge \varphi (\mathbf{x})$ for *all* points $\mathbf{x}\in \varOmega $.
- *Obstacle SOR*: The iterative algorithm for solving the linear system of the obstacle problem is implemented based on one of simplest iterative algorithms, the successive over-relaxation (SOR) method. Convergence of the obstacle SOR method is analyzed and compared with modern sophisticated methods. We also suggest an effective way to set the optimal relaxation parameter *ω*. Our simple obstacle SOR method with the optimal parameter performs better than state-of-the-art methods in both accuracy and efficiency.
- *Effective numerical methods for nonlinear problems*: For the nonlinear obstacle problem (1.1), a method of *gradient-weighting* is introduced to solve the problem more conveniently and efficiently. In particular, the suggested numerical schemes for the gradient-weighting problem produce an algebraic system of a symmetric and diagonally dominant *M*-matrix of which the main diagonal entries are all the same positive constant. Thus the resulting system is easy to implement and presumably converges fast; as one can see from Section 5, the obstacle SOR algorithm for nonlinear problems converges in a similar number of iterations as for linear problems.

The article is organized as follows. The next section presents a brief review for state-of-the-art methods for elliptic obstacle problems focusing the one in [14]. Also, accuracy deterioration of the numerical solution (underestimation) is discussed by exemplifying an obstacle problem in 1D where the mesh grid does not match with edges of the free boundary. In Section 3, the SOR is applied for both linear and nonlinear problems and analyzed for convergence; the limits of iterates are proved to satisfy discrete obstacle problems. A method of gradient-weighting and second-order FD schemes are introduced for nonlinear problems. An effective strategy is suggested to find the optimal relaxation parameter. Section 4 introduces subgrid FD schemes near the free boundary in order to reduce accuracy deterioration of the numerical solution. In Section 5, various numerical examples are included to verify the claims we just made. Section 6 concludes the article summarizing our experiments and findings.

## Preliminaries

As preliminaries, we first present a brief review for state-of-the-art methods for elliptic obstacle problems and certain accuracy issues related to the free boundary.

### State-of-the-art methods for elliptic obstacle problems

This subsection summarizes state-of-the-art methods for elliptic obstacle problems focusing on the *primal-dual method incorporating* $L^{1}$ *-like penalty term* (PDL1P) studied by Zosso *et al.* [14]. Primal-dual splitting methods have a great deal of attention, particularly in the context of total variation (TV) minimization and $L^{1}$-type problems in image processing [15–19].

In the literature of optimization problems, one of common practices is to reformulate a constrained optimization problem for a unconstrained problem by incorporating the constraint as a penalty term. Recently, Tran *et al.* [13] proposed the following minimization problem of a $L^{1}$-like penalty term:

2.1 $$ \min_{u} \int_{\varOmega }|\nabla u|^{2}+\mu(\varphi -u)_{+}, \quad\text{s.t. } u|_{\varGamma }=f, $$

where *μ* is a Lagrange multiplier and $(\cdot)_{+}=\max(\cdot,0)$. It is shown that, for sufficiently large but finite *μ*, the minimizer of the unconstrained problem (2.1) is also the minimizer of the original, constrained problem (1.2).

The PDL1P [14] is a hybrid method which combines primal-dual splitting algorithm and the $L^{1}$-like penalty method in (2.1); it can be summarized as follows.

2.2 $$ \left [ \textstyle\begin{array}{l} \mbox{Initialize } u^{0},\overline{u}^{0},p^{0}\leftarrow0. \\ \mbox{Repeat}\\ \quad \textstyle\begin{array}{ll} \mbox{(a)} & p^{n+1}=(p^{n}+r_{1} \nabla_{h}\overline{u}^{n})/(1+r_{1}),\\ \mbox{(b)} & u^{*} = u^{n}+r_{2}\nabla_{h}\cdot p^{n+1}, \\ \mbox{(c)} & u^{n+1}=\mathcal{P}_{\varphi }(u^{*}),\\ \mbox{(d)} & \overline{u}^{n+1}=2u^{n+1}-u^{n}, \end{array}\displaystyle \\ \mbox{until } \|u^{n+1}-u^{n}\|_{\infty}< \varepsilon , \end{array}\displaystyle \right . \quad\mbox{(PDL1P~[14])} $$

where $\nabla_{h}$ denotes the numerical approximation of the gradient ∇, associated with the mesh size *h*, $r_{1}$ and $r_{2}$ are constants to be determined, $p^{n}$ is the dual variable representing the gradient of the primal variable ($u^{n}$), and $u^{*}$ is an intermediate solution. Here $\mathcal{P}_{\varphi }$ is an obstacle projection defined by

2.3 $$ \mathcal{P}_{\varphi } \bigl(u^{*} \bigr) (\mathbf{x})= \left \{ \textstyle\begin{array}{l@{\quad}l} f(\mathbf{x}) & \mbox{if }\mathbf{x}\in \varGamma , \\ u^{*}(\mathbf{x})+r_{2}\mu& \mbox{if }\mathbf{x}\notin \varGamma \text{ and } u^{*}(\mathbf{x})< \varphi (\mathbf{x})-r_{2}\mu, \\ \varphi (\mathbf{x}) & \mbox{if }\mathbf{x}\notin \varGamma \text{ and }\varphi (\mathbf{x})-r_{2}\mu\le u^{*}(\mathbf{x})\le \varphi (\mathbf{x}), \\ u^{*}(\mathbf{x}) &\mbox{otherwise}. \end{array}\displaystyle \right . $$

The above algorithm can be implemented effectively. It follows from (2.2)(a) that

2.4 $$ \nabla_{h}\cdot p^{n+1}=\frac{\nabla_{h}\cdot p^{n}+r_{1} \Delta_{h}\overline{u}^{n}}{1+r_{1}}, $$

where $\Delta_{h}$ is the discrete Laplacian. Thus $S^{n+1}\equiv\nabla_{h}\cdot p^{n+1}$ can be considered as a variable and updated in each iteration, averaging its previous iterate $S^{n}$ and $\Delta_{h}\overline{u}^{n}$ as in (2.4). As analyzed in [14], PDL1P (away from the obstacle) can be compared to either the forward Euler (explicit) scheme for discrete heat equation or a three-level time stepping method for a damped acoustic wave equation, where $r_{1}r_{2}$ plays the role of the time-step size. PDL1P converges when

2.5 $$ r_{1}r_{2}\|\Delta_{h}\|\le1, $$

where $\|\Delta_{h}\|$ is the operator/induced norm of the discrete Laplacian $\Delta_{h}$ (=8, when the mesh size $h=1$). The authors in [14] claimed that ‘[Their] results achieve state-of-the-art precision in much shorter time; the speed up is one-two orders of magnitude with respect to the method in [13], and even larger compared to older methods [20–22].’ Thus, in this article our suggested method would be compared mainly with PDL1P (the best-known method), in order to show its superiority.

### Accuracy issues

**Figure 1:**
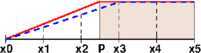
**A non-matching grid: The true solution** ***u*** **(red solid curve) and the numerical solution on the non-matching grid** $\pmb{u_{h}}$ **(blue dashed curve).** Here the obstacle is the shaded region, $u(x_{0})=u_{h}(x_{0})=\varphi (x_{0})=0$, and $u(x_{5})=u_{h}(x_{5})=\varphi (x_{5})=1$.

The solution of obstacle problems must lie on or over the obstacle ($u\ge \varphi $), which is also one of requirements for numerical solutions. For FD methods and *finite element* (FE) methods for the obstacle problem (1.3), for example, this requirement can easily be violated when edges of the free boundary does not match with mesh grids. See Figure 1, where the shaded rectangle indicates the obstacle defined on one-dimensional (1D) interval $[x_{0},x_{5}]$:

2.6 $$ \varphi (x)=\left \{ \textstyle\begin{array}{l@{\quad}l} 0 &\mbox{if }x_{0}\le x< p,\\ 1 &\mbox{if }p\le x\le x_{5}, \end{array}\displaystyle \right . $$

which is not matching with the mesh grids $\{x_{i}: x_{i}=i\cdot h_{x}, i=0,\ldots,5\}$. The figure shows the true solution *u* (red solid curve) and a numerical solution $u_{h}$ (blue dashed curve) of the linear obstacle problem (1.3) in 1D. The numerical solution is clearly underestimated and the magnitude of the error $|u_{h}-u|$ is maximized at $x=p$:

2.7 $$ \max_{x} \bigl|u_{h}(x)-u(x) \bigr| = \bigl|u_{h}(p\bigr)-u(p) \bigr| = \frac{x_{3}-p}{x_{3}-x_{0}}, $$

which is $\mathcal{O}(h_{x})$.

Let $C_{h}$ denote the numerical contact set:

2.8 $$ C_{h}\equiv\bigl\{ x\in \varOmega _{h}^{0}: u_{h}(x)=\varphi (x) \bigr\} , $$

where $\varOmega _{h}^{0}$ is the set of *interior* grid points. Define an interior grid point is a *neighboring point* if it is not in the contact set but one of its adjacent grid points is in the contact set. Let the set of neighboring points be called the *neighboring set* $N_{h}$. Then, for the example in Figure 1, $C_{h}=\{x_{3},x_{4}\}$ and $N_{h}=\{x_{2}\}$.

The accuracy of the numerical solution $u_{h}$ can be improved by applying a post-processing in which a subgrid FD method is applied at grid points in the neighboring set. For example, at $x=x_{2}$, $-u_{xx}$ can be approximated by employing nonuniform FD schemes over the grid points $[x_{1},x_{2},p]$, given as

2.9 $$ -u_{xx}(x_{2}) \approx\frac{2}{h_{x}^{2}} \biggl( -\frac{u_{1}}{1+r} +\frac{u_{2}}{r} -\frac{\varphi (p)}{r(1+r)} \biggr), $$

where $r=(p-x_{2})/h_{x}\in(0,1]$, and therefore numerical solution of $-u_{xx}=0$ at $x=x_{2}$ must satisfy

2.10 $$ u_{2}=\frac{r u_{1}+\varphi (p)}{1+r}. $$

As *r* is approaching 0 (*i.e.*, $(p-x_{2})$ becomes smaller proportionally), the obstacle value $\varphi (p)$ is more weighted. On the other hand, when $r=1$, $\varphi (p)=u_{3}$ and the scheme in (2.9) becomes the standard second-order FD scheme. Let the numerical solution *ũ* be obtained from

2.11 $$ \varphi (x_{j})\le \widetilde{u}_{j}= \left \{ \textstyle\begin{array}{l@{\quad}l} (r \widetilde{u}_{j-1}+\varphi (p))/(1+r) & \mbox{if }j=2,\\ (\widetilde{u}_{j-1}+\widetilde{u}_{j+1})/2 & \mbox{if }j=1,3,4, \end{array}\displaystyle \right . $$

where $\widetilde{u}_{0}=0$ and $\widetilde{u}_{5}=1$. Then it is not difficult to prove that *ũ* is *exactly* the same as the true solution *u* at all grid points (except numerical rounding error), regardless of the grid size $h_{x}$.

The above example has motivated the authors to develop an effective numerical algorithm for elliptic obstacle problems in 2D which detects the neighboring set of the free boundary, determines the subgrid proportions (*r*’s), and updates the solution for an improved accuracy using subgrid FD schemes. Here the main goal is to try to guarantee $u(\mathbf{x})\ge \varphi (\mathbf{x})$ for all $\mathbf{x}\in \varOmega $ (whether **x** is a grid point or not). Since it is often the case that the free boundary is determined only after solving the problem, the algorithm must be a post-process. Details are presented in Section 4.

## Obstacle relaxation methods

This section introduces and analyzes effective relaxation methods for solving (1.3) and its nonlinear problem as shown in (3.10) below.

### The linear obstacle problem

We will begin with second-order approximation schemes for $-\Delta u$. For simplicity, we consider a rectangular domain in $\mathbb{R}^{2}$, $\varOmega =(a_{x},b_{x})\times(a_{y},b_{y})$. Then the following second-order FD scheme can be formulated on the grid points:

3.1 $$ \mathbf{x}_{pq}:=(x_{p},y_{q}), \quad p=0,1,\ldots,n_{x}, q=0,1,\ldots,n_{y}, $$

where, for some positive integers $n_{x}$ and $n_{y}$,

$$x_{p}=a_{x}+p\cdot h_{x},\qquad y_{q}=a_{y}+q\cdot h_{y}; \quad h_{x}= \frac{b_{x}-a_{x}}{n_{x}}, h_{y}=\frac{b_{y}-a_{y}}{n_{y}}. $$

Let $u_{pq}=u(x_{p},y_{q})$. Then, at each of the interior points $\mathbf{x}_{pq}$, the five-point FD approximation of $-\Delta u$ reads

3.2 $$ -\Delta_{h} u_{pq}= \frac {-u_{p-1,q}+2u_{pq}-u_{p+1,q}}{h_{x}^{2}} + \frac{-u_{p,q-1}+2u_{pq}-u_{p,q+1}}{h_{y}^{2}}. $$

Multiply both sides of (3.2) by $h_{x}^{2}$ to have

3.3 $$ (-\Delta_{h} u_{pq}) h_{x}^{2} = \bigl(2+2 r_{xy}^{2} \bigr)u_{pq} -u_{p-1,q} -u_{p+1,q} -r_{xy}^{2} u_{p,q-1} -r_{xy}^{2} u_{p,q+1}, $$

where $r_{xy}=h_{x}/h_{y}$ and $u_{st}=f_{st}$ at boundary grid points $(x_{s},y_{t})$.

Now, consider the following Jacobi iteration for simplicity. Given an initialization $u^{0}$, find $u^{n}$ iteratively as follows.

#### Algorithm $\mathcal{L}_{J}$

3.4 $$ \textstyle\begin{array}{l} \mbox{For } n=1,2,\ldots\\ \quad\mbox{For } q=1:n_{y}-1 \\ \quad\mbox{For } p=1:n_{x}-1 \\ \quad\quad \textstyle\begin{array}{ll} \mbox{(a)} & u_{J,pq} = \frac{1}{2+2 r_{xy}^{2}} (u_{p-1,q}^{n-1} +u_{p+1,q}^{n-1} +r_{xy}^{2} u_{p,q-1}^{n-1} +r_{xy}^{2} u_{p,q+1}^{n-1} ); \\ \mbox{(b)} & u_{pq}^{n} =\max(u_{J,pq},\varphi_{pq}); \end{array}\displaystyle \\ \quad\mbox{end} \\ \quad\mbox{end} \\ \mbox{end} \end{array} $$

where $u^{n-1}_{st}=f_{st}$ at boundary grid points $(x_{s},y_{t})$.

Note that Algorithm $\mathcal{L}_{J}$produces a solution *u* of which the function value at a point is a simple average of four neighboring values, satisfying the constraint $u\ge\varphi$.

#### Theorem 1

*Let* *û* *be the limit of the iterates* $u^{n}$ *of Algorithm* $\mathcal{L}_{J}$. *Then* *û* *satisfies the FD discretization of* (1.3). *That is*,

3.5 $$ \textstyle\begin{array}{l@{\quad}l} \left . \textstyle\begin{array}{l} -\Delta_{h} \widehat{u}_{pq}\ge0, \\ \widehat{u}_{pq}\ge\varphi_{pq},\\ (-\Delta_{h} \widehat{u}_{pq})\cdot(\widehat{u}_{pq}-\varphi_{pq})=0, \end{array}\displaystyle \right \} & (x_{p},y_{q})\in \varOmega _{h}^{0}, \\ \widehat{u}_{st}=f_{st}, & (x_{s},y_{t})\in \varGamma _{h}, \end{array} $$

*where* $\varOmega _{h}^{0}$ *denotes the set of interior grid points and* $\varGamma _{h}$ *is the set of boundary grid points*.

#### Proof

It is clear to see from Algorithm $\mathcal{L}_{J}$that

$$\widehat{u}_{pq}\ge\varphi_{pq} \quad\text{for } (x_{p},y_{q}) \in \varOmega _{h}^{0} \quad \mbox{and} \quad \widehat{u}_{st}=f_{st} \quad\text{for } (x_{s},y_{t})\in \varGamma _{h}. $$

Let $\widehat{u}_{pq}=\varphi_{pq}$ at an interior point $(x_{p},y_{q})$. Then it follows from (3.4)(b) that

3.6 $$ \widehat{u}_{J,pq} = \frac{1}{2+2 r_{xy}^{2}} \bigl( \widehat{u}_{p-1,q} +\widehat{u}_{p+1,q} +r_{xy}^{2} \widehat{u}_{p,q-1} +r_{xy}^{2} \widehat{u}_{p,q+1} \bigr) \le\varphi_{pq}=\widehat{u}_{pq}, $$

which implies that

3.7 $$ 0\le\bigl(2+2 r_{xy}^{2} \bigr) \widehat{u}_{pq} -\widehat{u}_{p-1,q} -\widehat{u}_{p+1,q} -r_{xy}^{2} \widehat{u}_{p,q-1} -r_{xy}^{2} \widehat{u}_{p,q+1} = (-\Delta_{h} \widehat{u}_{pq}) \cdot h_{x}^{2}. $$

On the other hand, let $\widehat{u}_{pq}>\varphi_{pq}$ at $(x_{p},y_{q})$. Then, since $\widehat{u}_{pq}=\max(\widehat{u}_{J,pq},\varphi_{pq})$, we must have

3.8 $$ \widehat{u}_{pq} = \widehat{u}_{J,pq}, $$

which implies that $-\Delta_{h}\widehat{u}_{pq}=0$. This completes the proof. □

One can easily prove that the algebraic system obtained from (3.3) is irreducibly diagonally dominant and symmetric positive definite. Since its off-diagonal entries are all nonpositive, the matrix must be a Stieltjes matrix and therefore an M-matrix [23]. Thus relaxation methods of regular splittings (such as the Jacobi, the Gauss-Seidel (GS), and the successive over-relaxation (SOR) iterations) are all convergent and their limits are the same as *û* and therefore satisfy (3.5). In this article, variants of Algorithm $\mathcal{L}_{J}$for the GS and the SOR would be denoted, respectively, by $\mathcal{L}_{\mathrm{GS}}$ and $\mathcal{L}_{\mathrm{SOR}}$ $(\omega)$, where *ω* is an over-relaxation parameter for the SOR, $1<\omega<2$. For example, $\mathcal{L}_{\mathrm{SOR}}$ $(\omega)$ is formulated as

#### Algorithm $\mathcal{L}_{\mathrm{SOR}}$$(\omega)$

3.9 $$ \textstyle\begin{array}{l} \mbox{For } n=1,2,\ldots\\ \quad\mbox{For } q=1:n_{y}-1 \\ \quad\mbox{For } p=1:n_{x}-1 \\ \quad\quad \textstyle\begin{array}{ll} \mbox{(a)} & u_{\mathrm{GS},pq} = \frac{1}{2+2 r_{xy}^{2}} (u_{p-1,q}^{n} +u_{p+1,q}^{n-1} +r_{xy}^{2} u_{p,q-1}^{n} +r_{xy}^{2} u_{p,q+1}^{n-1} ); \\ \mbox{(b)} & u_{\mathrm{SOR},pq}=\omega\cdot u_{\mathrm{GS},pq} +(1-\omega)\cdot u_{pq}^{n-1};\\ \mbox{(c)} & u_{pq}^{n} =\max(u_{\mathrm{SOR},pq},\varphi_{pq}); \end{array}\displaystyle \\ \quad\mbox{end} \\ \quad\mbox{end} \\ \mbox{end} \end{array} $$

where $u^{n-1}_{st}=u^{n}_{st}=f_{st}$ at boundary grid points $(x_{s},y_{t})$.

Note that the right side of (3.9)(a) involves updated values wherever available. When $\omega=1$, Algorithm $\mathcal{L}_{\mathrm{SOR}}$ $(\omega)$ becomes Algorithm $\mathcal{L}_{\mathrm{GS}}$; that is, $\mathcal{L}_{\mathrm{SOR}}$ $(1)=\mathcal{L}_{\mathrm{GS}}$.

### The nonlinear obstacle problem

Applying the same arguments for the linear problem (1.3), the Euler-Lagrange equation for the nonlinear minimization problem (1.1) can be formulated as

3.10 $$ \textstyle\begin{array}{l@{\quad}l} \left . \textstyle\begin{array}{l} \mathcal{N}(u) \ge0, \\ u\ge\varphi,\\ \mathcal{N}(u)\cdot(u-\varphi)=0, \end{array}\displaystyle \right \} & \text{in }\varOmega , \\ u=f, & \text{on }\varGamma , \end{array} $$

where

3.11 $$ \mathcal{N}(u)=-\nabla\cdot\biggl(\frac{\nabla u}{\sqrt {1+|\nabla u|^{2}}} \biggr). $$

Thus the solution to the nonlinear problem (3.10) can be considered as a minimal surface satisfying the constraint given by the obstacle function *φ*.

Since $\sqrt{1+|\nabla u|^{2}}\ge1$, the nonlinear obstacle problem (3.10) can equivalently be formulated as

3.12 $$ \textstyle\begin{array}{l@{\quad}l} \left . \textstyle\begin{array}{l} \mathcal{M}(u) \ge0, \\ u\ge\varphi,\\ \mathcal{M}(u)\cdot(u-\varphi)=0, \end{array}\displaystyle \right \} & \text{in } \varOmega , \\ u=f, & \text{on }\varGamma , \end{array} $$

where

3.13 $$ \mathcal{M}(u)=-\sqrt{1+|\nabla u|^{2}} \nabla\cdot\biggl( \frac{\nabla u}{\sqrt{1+|\nabla u|^{2}}} \biggr). $$

Such a method of gradient-weighting will make algebraic systems simpler and better conditioned, as to be seen below. In order to introduce effective FD schemes for $\mathcal{M}(u)$, we first rewrite $\mathcal{M}(u)$ as

3.14 $$ \mathcal{M}(u)= - \bigl(\sqrt{1+|\nabla u|^{2}} \bigr)_{1} \biggl( \frac{u_{x}}{\sqrt{1+|\nabla u|^{2}}} \biggr)_{x} - \bigl(\sqrt {1+|\nabla u|^{2}} \bigr)_{2} \biggl(\frac{u_{y}}{\sqrt{1+|\nabla u|^{2}}} \biggr)_{y}, $$

where both $(\sqrt{1+|\nabla u|^{2}} )_{1}$ and $(\sqrt{1+|\nabla u|^{2}} )_{2}$ are the same as $\sqrt{1+|\nabla u|^{2}}$; however, they will be approximated in a slightly different way. The following numerical schemes are of second-order accuracy and specifically designed for the resulting algebraic system to be simpler and better conditioned.

For the FD scheme at the $(p,q)$th pixel, we first compute second-order FD approximations of $\sqrt{1+|\nabla u|^{2}}$ at $\mathbf{x}_{p-1/2,q} (W)$, $\mathbf{x}_{p+1/2,q} (E)$, $\mathbf{x}_{p,q-1/2} (S)$, and $\mathbf{x}_{p,q+1/2} (N)$:

3.15 $$ \begin{aligned} &d_{pq,W}= \bigl[1+(u_{pq}-u_{p-1,q})^{2}/h_{x}^{2} \\ &\hphantom{d_{pq,W}=} +(u_{p-1,q+1}+u_{p,q+1}-u_{p-1,q-1}-u_{p,q-1})^{2}/ \bigl(16h_{y}^{2}\bigr)\bigr]^{1/2}, \\ &d_{pq,E}= d_{p+1,q,W}, \\ &d_{pq,S}= \bigl[1+(u_{pq}-u_{p,q-1})^{2}/h_{y}^{2} \\ &\hphantom{d_{pq,S}=} +(u_{p+1,q}+u_{p+1,q-1}-u_{p-1,q}-u_{p-1,q-1})^{2}/ \bigl(16h_{x}^{2}\bigr)\bigr]^{1/2}, \\ &d_{pq,N}= d_{p,q+1,S}. \end{aligned} $$

Then the directional-derivative terms at the pixel point $\mathbf{x}_{pq}$ can be approximated by

3.16 $$ \begin{aligned} &\biggl(\frac{u_{x}}{\sqrt{1+|\nabla u|^{2}}} \biggr)_{x} (\mathbf{x}_{pq}) \approx\frac{1}{h_{x}^{2}} \biggl[ \frac{1}{d_{pq,W}}u_{p-1,q} +\frac{1}{d_{pq,E}}u_{p+1,q} - \biggl(\frac{1}{d_{pq,W}}+\frac{1}{d_{pq,E}} \biggr)u_{pq} \biggr], \\ &\biggl(\frac{u_{y}}{\sqrt{1+|\nabla u|^{2}}} \biggr)_{y} (\mathbf{x}_{pq}) \approx\frac{1}{h_{y}^{2}} \biggl[ \frac{1}{d_{pq,S}}u_{p,q-1} + \frac{1}{d_{pq,N}}u_{p,q+1} - \biggl( \frac{1}{d_{pq,S}}+\frac{1}{d_{pq,N}} \biggr)u_{pq} \biggr]. \end{aligned} $$

Now, we discretize the surface element as follows:

3.17 $$ \begin{aligned} & \bigl(\sqrt{1+|\nabla u|^{2}} \bigr)_{1}(\mathbf{x}_{pq}) \approx\biggl[ \frac{1}{2} \biggl(\frac{1}{d_{pq,W}}+\frac{1}{d_{pq,E}} \biggr) \biggr]^{-1} =\frac{2d_{pq,W}d_{pq,E}}{d_{pq,W}+d_{pq,E}}, \\ & \bigl(\sqrt{1+|\nabla u|^{2}} \bigr)_{2}( \mathbf{x}_{pq}) \approx\biggl[\frac{1}{2} \biggl(\frac{1}{d_{pq,S}}+ \frac{1}{d_{pq,N}} \biggr) \biggr]^{-1} =\frac{2d_{pq,S}d_{pq,N}}{d_{pq,S}+d_{pq,N}}, \end{aligned} $$

where the right-hand sides are harmonic averages of FD approximations of $\sqrt{1+|\nabla u|^{2}}$ in *x*- and *y*-coordinate directions, respectively. Then it follows from (3.14), (3.16), and (3.17) that

3.18 $$\begin{aligned} \mathcal{M}(u) (\mathbf{x}_{pq})\cdot h_{x}^{2} \approx{}& \bigl(2+2 r_{xy}^{2}\bigr) u_{pq}-a_{pq,W}u_{p-1,q}-a_{pq,E}u_{p+1,q} \\ & -r_{xy}^{2}a_{pq,S}u_{p,q-1}-r_{xy}^{2} a_{pq,N}u_{p,q+1}, \end{aligned}$$

where

3.19 $$ \begin{aligned} &a_{pq,W}=\frac{2 d_{pq,E}}{d_{pq,W}+d_{pq,E}}, \qquad a_{pq,E}=\frac{2 d_{pq,W}}{d_{pq,W}+d_{pq,E}}, \\ &a_{pq,S}=\frac{2 d_{pq,N}}{d_{pq,S}+d_{pq,N}}, \qquad a_{pq,N}=\frac{2 d_{pq,S}}{d_{pq,S}+d_{pq,N}}. \end{aligned} $$

Note that $a_{pq,W}+a_{pq,E}=a_{pq,S}+a_{pq,N}=2$. As for the linear problem, it is easy to prove that the algebraic system obtained from (3.18) is an M-matrix.

Given FD schemes for $\mathcal{M}(u)$ as in (3.18), the nonlinear obstacle problem (3.12) can be solved iteratively by the Jacobi iteration.

#### Algorithm $\mathcal{N}_{J}$

3.20 $$ \textstyle\begin{array}{l} \mbox{For } n=1,2,\ldots\\ \quad\mbox{For } q=1:n_{y}-1 \\ \quad\mbox{For } p=1:n_{x}-1 \\ \quad\quad \textstyle\begin{array}{ll} \mbox{(a)} & u_{J,pq} = \frac{1}{2+2 r_{xy}^{2}} (a_{pq,W}^{n-1}u_{p-1,q}^{n-1} +a_{pq,E}^{n-1}u_{p+1,q}^{n-1} +r_{xy}^{2} a_{pq,S}^{n-1}u_{p,q-1}^{n-1} +r_{xy}^{2} a_{pq,N}^{n-1}u_{p,q+1}^{n-1} ); \\ \mbox{(b)} & u_{pq}^{n} =\max(u_{J,pq},\varphi_{pq}); \end{array}\displaystyle \\ \quad\mbox{end} \\ \quad\mbox{end} \\ \mbox{end} \end{array} $$

where $u^{n-1}_{st}=f_{st}$ at boundary grid points $(x_{s},y_{t})$.

The superscript $(n-1)$ on the coefficients $a_{pq,D}$, $D=W, E, S, N$, indicate that they are obtained using the last iterate $u^{n-1}$. Algorithm $\mathcal{N}_{J}$produces a solution *u* of which the function value at a point is a *weighted* average of four neighboring values, satisfying the constraint $u\ge\varphi$. One can prove the following corollary, using the same arguments introduced in the proof of Theorem 1.

#### Corollary 1

*Let* *û* *be the limit of the iterates* $u^{n}$ *of Algorithm* $\mathcal{N}_{J}$. *Then* *û* *satisfies the FD discretization of* (3.12). *That is*,

3.21 $$ \textstyle\begin{array}{l@{\quad}l} \left . \textstyle\begin{array}{l} \mathcal{M}_{h}(\widehat{u})_{pq}\ge0, \\ \widehat{u}_{pq}\ge\varphi_{pq},\\ \mathcal{M}_{h}(\widehat{u})_{pq}\cdot(\widehat{u}_{pq}-\varphi_{pq})=0, \end{array}\displaystyle \right \} & (x_{p},y_{q})\in \varOmega _{h}^{0}, \\ \widehat{u}_{st}=f_{st}, & (x_{s},y_{t})\in \varGamma _{h}, \end{array} $$

*where* $\mathcal{M}_{h}(\widehat{u})_{pq}$ *denotes the FD scheme of* $\mathcal{M}(u)(\mathbf{x}_{pq})$ *as defined in* (3.18) *with* $u=\widehat{u}$.

Variants of Algorithm $\mathcal{N}_{J}$for the GS and the SOR can be formulated similarly as for the linear obstacle problem; they would be denoted respectively by $\mathcal{N}_{\mathrm{GS}}$ and $\mathcal{N}_{\mathrm{SOR}}$ $(\omega)$. In practice, such symmetric coercive optimization problems, the SOR methods are much more efficient than the Jacobi and Gauss-Seidel methods. We will exploit $\mathcal{L}_{\mathrm{SOR}}$ $(\omega)$ and $\mathcal{N}_{\mathrm{SOR}}$ $(\omega)$ for numerical comparisons with state-of-the-art methods, by setting the relaxation parameter *ω* optimal.

### The optimal relaxation parameter *ω̂*

Consider the standard Poisson equation with a Dirichlet boundary condition

3.22 $$ \begin{aligned} &{-}\Delta u=g \quad\mbox{in } \varOmega ,\\ &u=f \quad\mbox{on } \varGamma =\partial \varOmega , \end{aligned} $$

for prescribed functions *f* and *g*. Let $\varOmega =[0,1]^{2}$, for simplicity, and apply the second-order FD method for the second derivatives on a uniform grid: $h=h_{x}=h_{y}=1/(m+1)$, for some positive integer. The its algebraic system can be written as

3.23 $$ A\mathbf{u}= \mathbf{b}\in \mathbb{R}^{m^{2}}. $$

Then the theoretically optimal relaxation parameter for the SOR method can be determined as [23], Section 4.3,

3.24 $$ \widehat{\omega} = \frac{2}{1+\sqrt {1-\rho(T_{J})^{2}}}, $$

where $\rho(T_{J})$ is the spectral radius of the iteration matrix of the Jacobi method $T_{J}$. The iteration matrix $T_{J}$ can be explicitly presented as a block tridiagonal matrix

3.25 $$ T_{J}=\frac{1}{4} \operatorname{tridiag}(I_{m},B_{m},I_{m}), $$

where $I_{m}$ is the *m*-dimensional identity matrix and

$$B=\operatorname{tridiag}(1,0,1) =\left [ \textstyle\begin{array}{lllll} 0 & 1 & && \\ 1 & 0 & 1 & & \\ & \ddots& \ddots& \ddots\\ && 1 & 0 & 1 \\ &&& 1 & 0 \end{array}\displaystyle \right ] \in \mathbb{R}^{m\times m}. $$

For such a matrix $T_{J}$, it is well known that

3.26 $$ \rho(T_{J})=1-ch^{2}, \quad\mbox{for some } c>0. $$

Thus it follows from (3.24) and (3.26) that the optimal SOR parameter corresponding to the mesh size *h*, $\widehat{\omega}_{h}$, can be expressed as

3.27 $$ \widehat{\omega}_{h} =\frac{2}{1+\sqrt {1-(1-ch^{2})^{2}}} = \frac{2}{1+\sqrt{2ch^{2}-c^{2}h^{4}}} \approx\frac{2}{1+c_{0} h}, $$

where $c_{0}=\sqrt{2c}$. Hence, for general mesh size *h*, the corresponding optimal SOR parameter $\widehat{\omega}_{h}$ can be found as follows.

3.28 $$ \left \{ \textstyle\begin{array}{ll} \mbox{(a)} & \mbox{Determine $\widehat{\omega}_{h_{0}}$ for a prescribed mesh size $h=h_{0}$, } \textit{heuristically}.\\ \mbox{(b)} & \mbox{Find $c_{0}$ by solving (3.27) for $c_{0}$:}\\ & \quad c_{0}=(2/\widehat{\omega}_{h_{0}}-1)/h_{0}. \\ \mbox{(c)} & \mbox{Use (3.27) with the above $c_{0}$ to determine $\widehat{\omega}_{h}$ for general $h$.} \end{array}\displaystyle \right . $$

It is often the case that the calibration (3.28)(a)-(3.28)(b) can be carried out with a small problem, *i.e.*, with $h_{0}$ of a very low resolution.

## Subgrid FD schemes for the free boundary: a post-process

This section describes subgrid FD schemes for the free boundary, focusing on the linear obstacle problem; the arguments to be presented can be applied the same way for nonlinear problems. Again, we assume for simplicity that $h=h_{x}=h_{y}$.

Let *û* be the numerical solution of an obstacle problem. Then it would satisfy the discrete obstacle problem (3.5), particularly $\widehat{u}_{pq}\ge\varphi_{pq}$ at all (interior) grid points $\mathbf{x}_{pq}\in \varOmega _{h}^{0}$. However, when the mesh grid is not matching with the free boundary, the obstacle constraint $\widehat{u}\ge \varphi $ may not be satisfied at all points $\mathbf{x}\in \varOmega $. This implies that when the mesh is not fine enough, the numerical solution can be underestimated near the free boundary, as shown in Figure 1 in Section 2.2. Note that the error introduced by non-matching grids is in $\mathcal{O}(h)$, while the numerical truncation error is in $\mathcal{O}(h^{2})$ for second-order FD schemes. That is, the underestimation is in $\mathcal{O}(h)$, which can be much larger than the truncation error. The strategy below can be considered as a post-processing algorithm designed in order to reduce the underestimation without introducing a mesh refinement. The post-processing algorithm consists of three steps: (a) finding the numerical contact set and the neighboring set, (b) subgrid determination of the free boundary, and (c) nonuniform FD schemes on the neighboring set.

### The contact set and the neighboring set

Finding the numerical contact set is an easy task. Let *û* and *φ* be the numerical solution and the lower obstacle, respectively. Then, for example, the characteristic set of contact points $C_{h}$ can be determined as follows.

4.1 $$ \left [ \textstyle\begin{array}{l} C_{h}=\widehat{u}-\varphi; \\ \mbox{if } C_{h}(\mathbf{x}_{pq})>0, \quad \mbox{then } C_{h}(\mathbf{x}_{pq})=1; \quad\mbox{for all points $\mathbf{x}_{pq}$}; \\ C_{h} = \mathbf{1}-C_{h}. \end{array}\displaystyle \right . $$

As defined in Section 2.2, an interior grid point is a neighboring point when it is not in the contact set but one of its adjacent grid points is in the contact set. Thus the neighboring points can be found more effectively as follows. Visit each point in the contact set; if any one of its four adjacent points is not in the contact set, then the non-contacting point is a neighboring point. The set of all neighboring points is the neighboring set $N_{h}$.

### Subgrid determination of the free boundary

**Figure 2:**
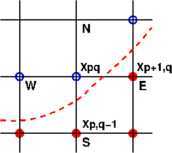
**Contact points (red solid circle) and neighboring points (blue open circle).** The red dashed curve indicates a possible free boundary.

Let $\mathbf{x}_{pq}$ be a neighboring point with two of its adjacent points are contact points ($C_{h}(p+1,q)=C_{h}(p,q-1)=1$), as in Figure 2. Then we may assume that the *real* free boundary passes somewhere between the contact points and the neighboring points. We will suggest an effective strategy for the determination of the free boundary in subgrid level.

We first focus on the horizontal line segment connecting $\mathbf{x}_{pq}$ and $\mathbf{x}_{p+1,q}$ in the east (E) direction. Define

4.2 $$ \mathbf{x}_{E}(r)=(1-r)\mathbf{x}_{pq}+r \mathbf{x}_{p+1,q}, \quad r\in[0,1]. $$

Then the corresponding linear interpolation between $\mathbf{u}_{pq}$ and $\mathbf{u}_{p+1,q}$ over the line segment is formulated as

4.3 $$ L_{E}(r)=(1-r)\mathbf{u}_{pq}+r \mathbf{u}_{p+1,q}, \quad r\in[0,1]. $$

Let

4.4 $$ F_{E}(r)=\varphi \bigl(\mathbf{x}_{E}(r) \bigr)-L_{E}(r), \quad r\in[0,1]. $$

Since $\mathbf{x}_{pq}$ and $\mathbf{x}_{p+1,q}$ are a neighboring point and a contact point, respectively, we have

4.5 $$ F_{E}(0)< 0\quad \mbox{and}\quad F_{E}(1)=0. $$

If the free boundary passes between $\mathbf{x}_{pq}$ and $\mathbf{x}_{p+1,q}$, then there must exist $r\in(0,1)$ such that $F_{E}(r)>0$. Let $r_{E}$ be such that $\mathbf{x}_{E}(r_{E})$ represents the intersection between the line segment $\mathbf{x}_{E}(\cdot)$ and the free boundary. Then it can be approximated as follows.

4.6 $$ r_{E} = \max_{r\in(0,1]} F_{E}(r). $$

The maximization problem in (4.6) can be solved easily (using the Newton method, for example), when the obstacle is defined as a smooth function. A more robust method can be formulated as a combination of a line search algorithm and the bisection method.

4.7 $$ \textstyle\begin{array}{l} \mbox{set $k_{0}$, $k_{1}$;}\\ r_{E}=1;\quad F_{\max}=0;\\ \mbox{for } k=1:k_{0}-1 \quad\mbox{\% line search}\\ \quad\mbox{if } F_{E}(k/k_{0})> F_{\max}\\ \quad\quad r_{E}=k/k_{0}; \quad F_{\max}=F_{E}(k/k_{0});\\ \quad\mbox{end}\\ \mbox{end}\\ \mbox{if } r_{E}< 1 \quad\mbox{\% refine it through bisection}\\ \quad r_{b}=1/k_{0};\\ \quad\mbox{for } k=1:k_{1} \\ \quad\quad r_{b}=r_{b}/2;\\ \quad\quad\mbox{if } F_{E}(r_{E}-r_{b})> F_{\max}\\ \quad\quad\quad r_{E}=r_{E}-r_{b}; \quad F_{\max}=F_{E}(r_{E}-r_{b});\\ \quad\quad\mbox{end}\\ \quad\quad\mbox{if } F_{E}(r_{E}+r_{b})> F_{\max}\\ \quad\quad\quad r_{E}=r_{E}+r_{b}; \quad F_{\max}=F_{E}(r_{E}+r_{b});\\ \quad\quad\mbox{end}\\ \quad\mbox{end}\\ \quad B_{E}=\varphi (\mathbf{x}_{E}(r_{E}));\\ \mbox{end} \end{array} $$

*Remarks*

- The last evaluation of *φ* (and saving) is necessary for the nonuniform FD schemes on the neighboring set, which will be discussed in Section 4.3. The quantity $B_{E}$ will be used as the Dirichlet value on the free boundary.
- For other directions *D* (=*W*, *S*, or *N*), one can define corresponding difference functions $F_{D}$ as shown in (4.2)-(4.4) for $D=E$. Then $r_{D}$ can be obtained by applying (4.7) with $F_{E}$ being replaced with $F_{D}$. When the adjacent point $\mathbf{x}_{D}(1)$ is not a contact point, you may simply set $r_{D}=1$. Thus each neighboring point produces an array of four values $[r_{W},r_{E},r_{S},r_{N}]$ and free boundary values for the directions *D* where $r_{D}<1$.
- Assuming that (4.6) has a unique solution and the obstacle is given as a smooth function, the maximum error for the detection of the free boundary using (4.7) is
  4.8 $$ \biggl(\frac{1}{k_{0}}\cdot\frac {1}{2^{k_{1}}} \biggr) h, $$
  where *h* is mesh size. It has been numerically verified that the choice $(k_{0},k_{1})=(10,4)$ is enough for an accurate detection of the free boundary, for which the upper bound of the error becomes $h/160=0.00625 h$.

### Nonuniform FD schemes on the neighboring set

Let $\mathbf{x}_{pq}=(x_{p},y_{q})$ be a neighboring point. Then $r_{pq,D}\in(0,1]$ would be available for each $D\in\{W,E,S,N\}$; $B_{pq,D}$ is also available for $r_{D}<1$. Thus the FD scheme for $-u_{xx}(\mathbf{x}_{pq})$ can be formulated over three points $\{(x_{p}-r_{pq,W} h_{x},y_{q}),(x_{p},y_{q}),(x_{p}+r_{pq,E} h_{x},y_{q})\}$ as follows.

4.9 $$ -u_{xx}(\mathbf{x}_{pq}) \approx\frac{2}{h_{x}^{2}} \biggl( -\frac{u_{pq,W}}{r_{pq,W}(r_{pq,W}+r_{pq,E})} +\frac {u_{pq}}{r_{pq,W}\cdot r_{pq,E}} -\frac {u_{pq,E}}{r_{pq,E}(r_{pq,W}+r_{pq,E})} \biggr), $$

where

$$u_{pq,W} = \left \{ \textstyle\begin{array}{l@{\quad}l} u_{p-1,q} &\mbox{if }r_{pq,W}=1,\\ B_{pq,W} &\mbox{if }r_{pq,W}< 1, \end{array}\displaystyle \right . \qquad u_{pq,E} = \left \{ \textstyle\begin{array}{l@{\quad}l} u_{p+1,q} &\mbox{if }r_{pq,E}=1,\\ B_{pq,E} &\mbox{if }r_{pq,E}< 1. \end{array}\displaystyle \right . $$

Similarly, the FD scheme for $-u_{yy}(\mathbf{x}_{pq})$ can be formulated over three points in the *y*-direction $\{(x_{p},y_{q}-r_{pp,S} h_{y}),(x_{p},y_{q}),(x_{p},y_{q}+r_{pp,N} h_{y})\}$:

4.10 $$ -u_{yy}(\mathbf{x}_{pq}) \approx\frac{2}{h_{y}^{2}} \biggl( -\frac{u_{pq,S}}{r_{pq,S}(r_{pq,S}+r_{pq,N})} +\frac {u_{pq}}{r_{pq,S}\cdot r_{pq,N}} -\frac {u_{pq,N}}{r_{pq,N}(r_{pq,S}+r_{pq,N})} \biggr), $$

where

$$u_{pq,S} = \left \{ \textstyle\begin{array}{l@{\quad}l} u_{p,q-1} &\mbox{if }r_{pq,S}=1,\\ B_{pq,S} &\mbox{if }r_{pq,S}< 1, \end{array}\displaystyle \right . \qquad u_{pq,N} = \left \{ \textstyle\begin{array}{l@{\quad}l} u_{p,q+1} &\mbox{if }r_{pq,N}=1,\\ B_{pq,N} &\mbox{if }r_{pq,N}< 1. \end{array}\displaystyle \right . $$

Thus, the post-processing algorithm of the obstacle SOR (3.9), $\mathcal{L}_{\mathrm{SOR}}(\omega)$, can be formulated by replacing the two terms in the right side of (3.2) with the right sides of (4.9) and (4.10), and computing $u_{\mathrm{GS},pq}$ in (3.9.a) correspondingly at all neighboring points.

## Numerical experiments

In this section, we apply the obstacle SOR method and the post-processing schemes to various obstacles to verify their effectiveness and accuracy. We mainly concern 2-D obstacle problems of Dirichlet boundary conditions. The algorithms are implemented, for both one and double obstacles, in Matlab and carried out on a Desktop computer of an Intel i5-3450S 2.80 GHz processor. The optimal relaxation parameter is calibrated with the lowest resolution to find a constant $c_{0}$ (3.28) and the constant is used for all other cases. For a comparison purpose, we implemented a state-of-the-art method, PDL1P [14], and its parameters ($r_{1}$ and $r_{2}$ in (2.2)) are found heuristically for cases where the parameters are not suggested in [14]. The iterations are stopped when the maximum difference of consecutive iterates becomes smaller than the tolerance *ε*:

5.1 $$ \bigl\| u^{n}-u^{n-1}\bigr\| _{\infty}< \varepsilon , $$

where $\varepsilon =10^{-6}$ mostly; Section 5.3 uses $\varepsilon =10^{-7}$ for an accurate estimation of the error. For all examples, the numerical solution is initialized from *φ* (the lower obstacle) and the boundary condition *f*.

5.2 $$ u^{0}(\mathbf{x}) = \left \{ \textstyle\begin{array}{l@{\quad}l} \varphi (\mathbf{x}) &\mbox{if }\mathbf{x}\in \varOmega _{h}^{0},\\ f(\mathbf{x}) &\mbox{if }\mathbf{x}\in \varGamma _{h}. \end{array}\displaystyle \right . $$

### Linear obstacle problems

We first consider a non-smooth obstacle $\varphi_{1}:\varOmega \rightarrow \mathbb{R}$ with $\varOmega =[0,1]^{2}$, defined by

5.3 $$ \varphi_{1}(x,y)=\left \{ \textstyle\begin{array}{l@{\quad}l} 5 & \mbox{if $|x-0.6|+|y-0.6|< 0.04$}, \\ 4.5 & \mbox{if $(x-0.6)^{2}+(y-0.25)^{2}< 0.001$}, \\ 4.5 & \mbox{if $y=0.57$ and $0.075< x< 0.13$}, \\ 0 & \mbox{otherwise}. \end{array}\displaystyle \right . $$

**Figure 3:**
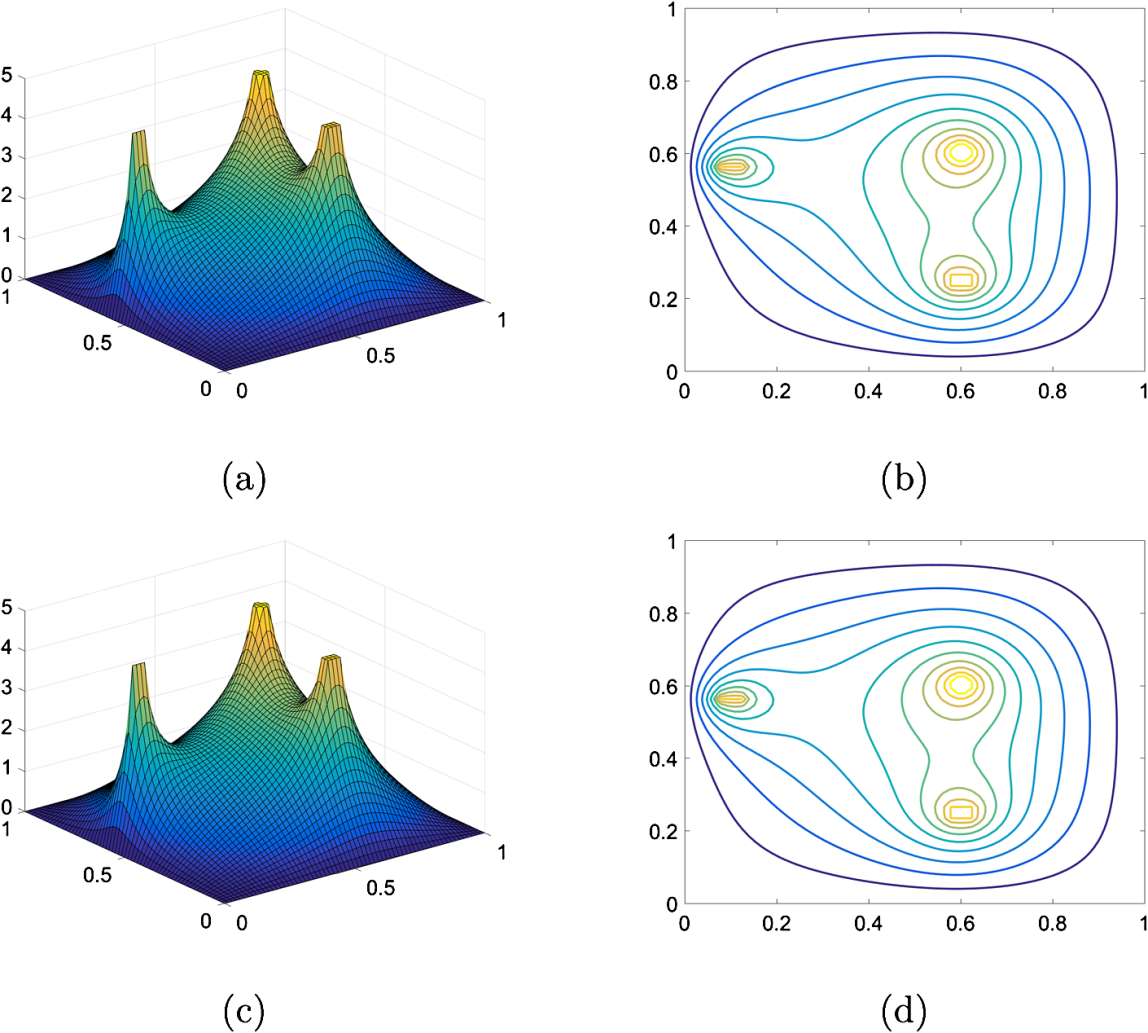
**Solutions to the linear problem for the obstacle** $\pmb{\varphi _{1}}$ **(** **5.3** **) at resolution** $\pmb{64\times64}$ **.** **(a)** The numerical solution by the PDL1P, **(b)** its contour plot, **(c)** the numerical solution by the obstacle SOR, and **(d)** its contour plot.

We solve the linear obstacle problem varying resolutions. The tolerance is set $\varepsilon =10^{-6}$ hereafter except for examples in Section 5.3. Table 1 presents the number of iterations and CPU (the elapsed time, measured in second) for the linear problem of the non-smooth obstacle (5.3). One can see from the table that our suggested method requires less iterations and converges about one order faster in the computation time than the PDL1P, a state-of-the-art method. We have also implemented the primal-dual hybrid gradient (PDHD) algorithm in [15, 18, 19] for obstacle problems. The PDL1P turns out to be a simple adaptation of the PDHD and their performances are about the same, particularly when *μ* is set large. For the resolution $64\times64$, Figure 3 depicts the numerical solutions of the PDL1P and the obstacle SOR and their contour lines. For this example, both the PDL1P and the obstacle SOR resulted in almost identical solutions.

**Table 1:** **The number of iterations and CPU for the linear problem of the non-smooth obstacle** $\pmb{\varphi _{1}}$ **(** **5.3** **)**

$\boldsymbol{(\varepsilon =10^{-6})}$	**PDL1P**		**Obstacle SOR**	
**Resolution**	**Iter**	**CPU**	**Iter**	**CPU**
32 × 32	1,284	0.13	84	0.005
64 × 64	1,744	0.71	148	0.03
128 × 128	2,111	3.58	268	0.22
256 × 256	2,097	14.09	525	1.70

For PDL1P [14], set *μ* = 10^8^, $r_{1}=0.01$, and $r_{2}=12.5$.

As the second example, we consider the radially symmetric obstacle $\varphi_{2}:\varOmega \rightarrow \mathbb{R}$ with $\varOmega =[-2,2]^{2}$ defined by

5.4 $$ \varphi_{2}(r)=\left \{ \textstyle\begin{array}{l@{\quad}l} \sqrt{1-r^{2}} & \mbox{if $r\leq r^{\ast}$}, \\ -1 & \mbox{otherwise}, \end{array}\displaystyle \right . $$

where $r^{\ast}=0.6979651482233\ldots$ , the solution of

5.5 $$ \bigl(r^{\ast} \bigr)^{2} \bigl(1-\log \bigl(r^{\ast}/2 \bigr) \bigr)=1. $$

For the obstacle $\varphi _{2}$, the analytic solution to the linear obstacle problem can be defined as

**Figure 4:**
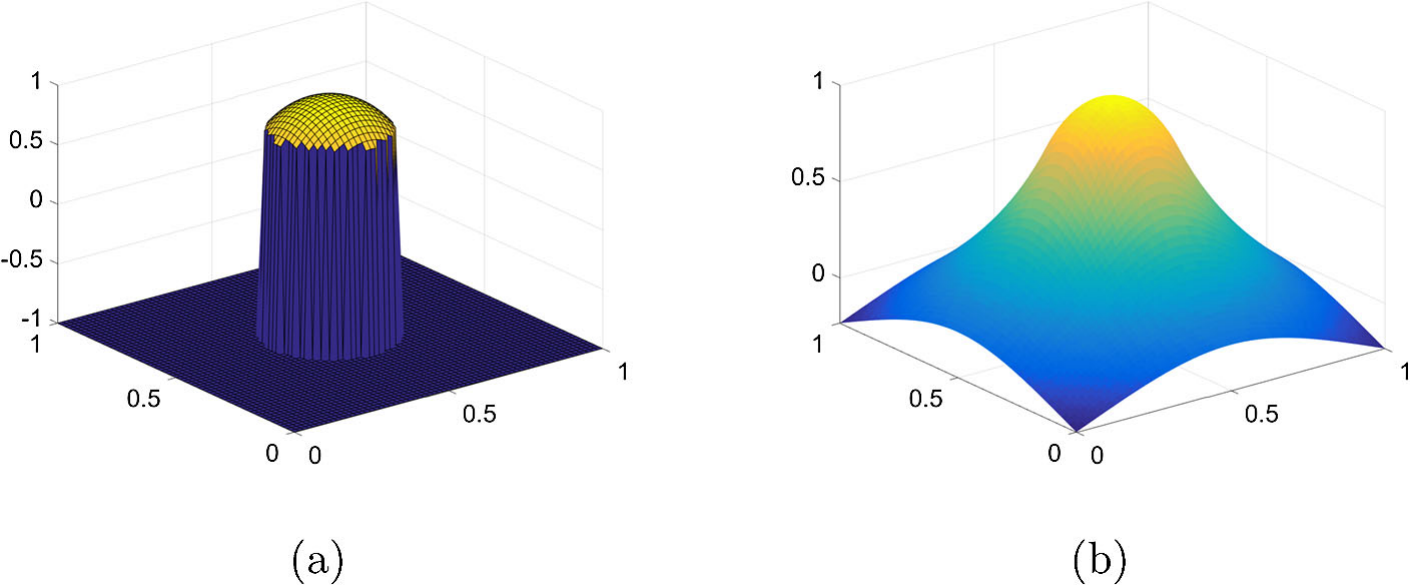
**The true solution to the linear problem for the obstacle** $\pmb{\varphi _{2}}$ **(** **5.4** **) at resolution** $\pmb{64\times64}$ **.** **(a)** The obstacle $\varphi _{2}$ and **(b)** the true solution $u^{*}$ (5.6)

5.6 $$ u^{\ast}(r)=\left \{ \textstyle\begin{array}{l@{\quad}l} \sqrt{1-r^{2}} & \mbox{if $r\leq r^{\ast}$}, \\ -(r^{\ast})^{2}\ln(r/2)/\sqrt{1-(r^{\ast})^{2}} & \mbox{otherwise}, \end{array}\displaystyle \right . $$

when the boundary condition is set appropriately using $u^{*}$. See Figure 4, in which we give plots of $\varphi _{2}$ and the true solution $u^{*}$.

**Figure 5:**
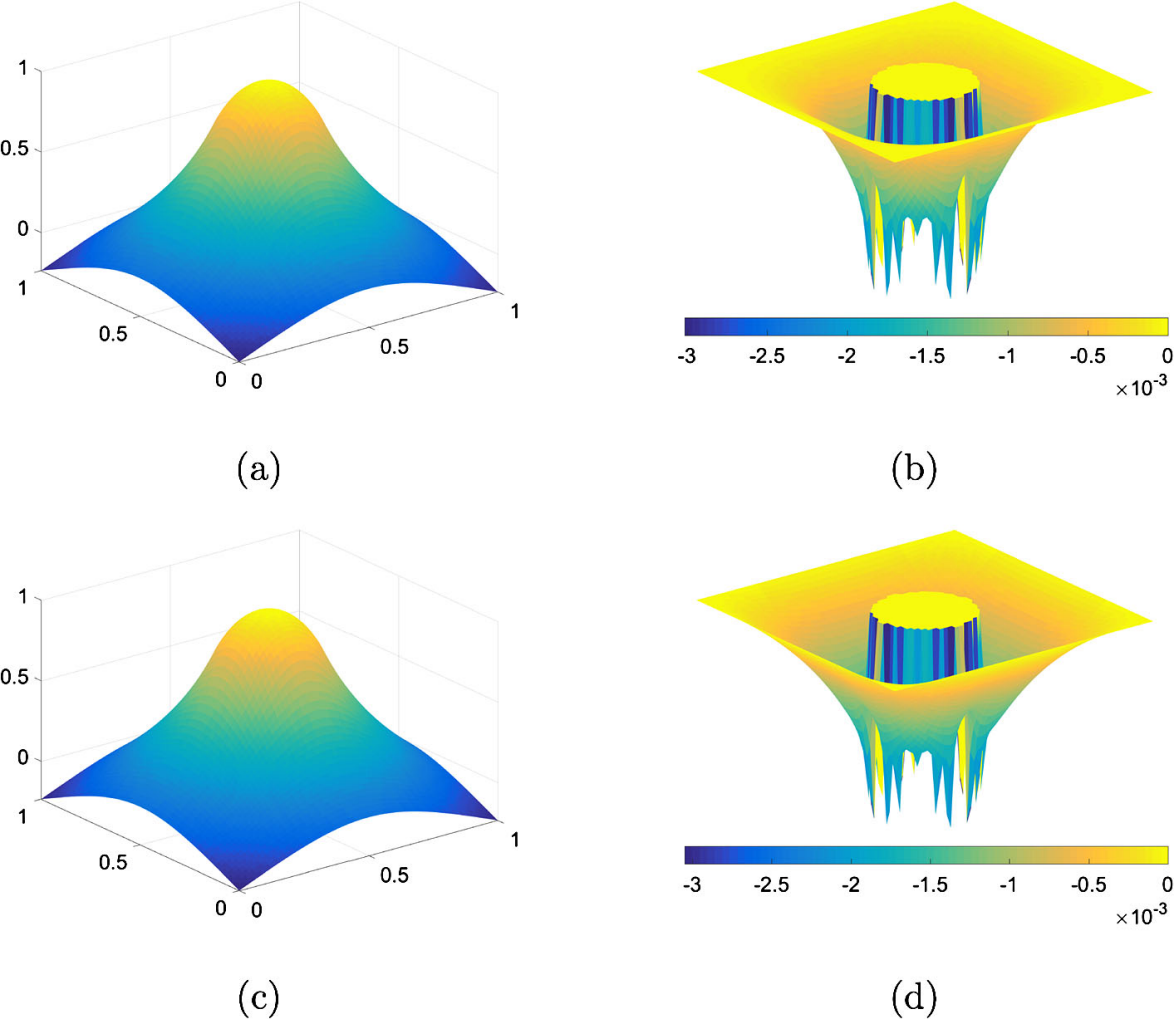
**Numerical solutions** $\pmb{u_{h}}$ **and errors** $\pmb{u_{h}-u^{*}}$ **at the** $\pmb{64\times64}$ **resolution.** **(a)**-**(b)** by the PDL1P and **(c)**-**(d)** by the obstacle SOR.

In Table 2, we compare performances of the PDL1P and the obstacle SOR applied for the linear obstacle problem with (5.4). The PDL1P uses the parameters suggested in [14] ($\mu=0.1$, $r_{1}=0.008$, $r_{2}=15.625$). As one can see from the table, our suggested method takes about one order less CPU time than the PDL1P for the computation of the numerical solution. In Figure 5, we show the numerical solutions $u_{h}$ and the errors $u_{h}-u^{*}$ produced by the PDL1P and the obstacle SOR at the $64\times64$ resolution. The solutions are almost identical and the errors are nonpositive. This implies that the numerical solutions of the obstacle problem are underestimated.

**Table 2:** $\pmb{L^{\infty}}$ **-errors, the number of iterations, and the CPU for linear obstacle problem with** $\pmb{\varphi _{2}}$ **(** **5.4** **)**

$\boldsymbol{(\varepsilon =10^{-6})}$	**PDL1P**		**Obstacle SOR**	
**Resolution**	$\boldsymbol{L^{\infty}}$ **-error**	**Iter (CPU)**	$\boldsymbol{L^{\infty}}$ **-error**	**Iter (CPU)**
32 × 32	8.91⋅10^−3^	715 (0.09)	8.69⋅10^−3^	62 (0.02)
64 × 64	3.01⋅10^−3^	1,340 (0.60)	3.05⋅10^−3^	122 (0.04)
128 × 128	7.66⋅10^−4^	1,971 (3.51)	7.64⋅10^−4^	244 (0.22)
256 × 256	1.86⋅10^−4^	2,072 (14.85)	1.88⋅10^−4^	489 (1.63)

The PDL1P uses the parameters suggested in [14] (*μ* = 0.1, $r_{1}=0.008$, $r_{2}=15.625$).

As a more general obstacle problem, we consider the elastic-plastic torsion problem in [22]. The problem is to find the equilibrium position of the membrane between two obstacles *φ*, *ψ* that a force *v* is acting on:

5.7 $$ \min_{u} \int_{\varOmega } |\nabla u|^{2}\, d\mathbf{x}- \int_{\varOmega } uv\, d\mathbf{x}, \quad\text{s.t. } \psi\ge u\ge\varphi \text{ in }\varOmega , u=f \text{ on }\varGamma . $$

Let $\varOmega =[0,1]^{2}$ and the problem consist of two obstacles $\varphi_{3}:\varOmega \rightarrow \mathbb{R}$, $\psi_{3}:\varOmega \rightarrow \mathbb{R}$ and the force $v:\varOmega \rightarrow \mathbb{R}$ defined by $\varphi_{3}(x,y)=-\operatorname{dist}(x,\partial \varOmega )$, $\psi _{3}(x,y)=0.2$ and

5.8 $$ v(x,y)=\left \{ \textstyle\begin{array}{l@{\quad}l} 300 & \mbox{if $(x,y)\in S=\{(x,y): |x-y|\leq0.1 \wedge x\leq 0.3\}$,} \\ -70e^{y}g(x) & \mbox{if $x\le1-y$ and $(x,y)\notin S$,} \\ 15e^{y}g(x) & \mbox{if $x> 1-y$ and $(x,y)\notin S$,} \end{array}\displaystyle \right . $$

where

5.9 $$ g(x)=\left \{ \textstyle\begin{array}{l@{\quad}l} 6x & \mbox{if }0\le x\le1/6, \\ 2(1-3x) & \mbox{if }1/6< x\le1/3, \\ 6(x-1/3) & \mbox{if }1/3< x\le1/2, \\ 2(1-3(x-1/3)) & \mbox{if }1/2< x\le2/3, \\ 6(x-2/3) & \mbox{if }2/3< x\le5/6, \\ 2(1-3(x-2/3)) & \mbox{if }5/6< x\le1. \end{array}\displaystyle \right . $$

**Figure 6:**
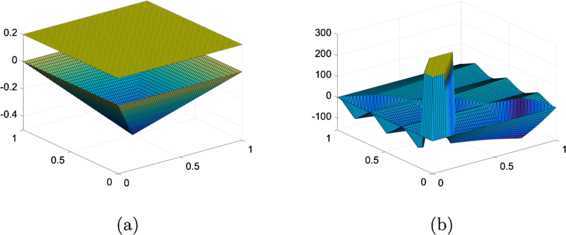
**Elastic-plastic torsion problem.** **(a)** The obstacles ($\psi_{3}$ and $\varphi _{3}$) and **(b)** the force *v*.

See Figure 6, where the obstacles and the force are depicted.

**Figure 7:**
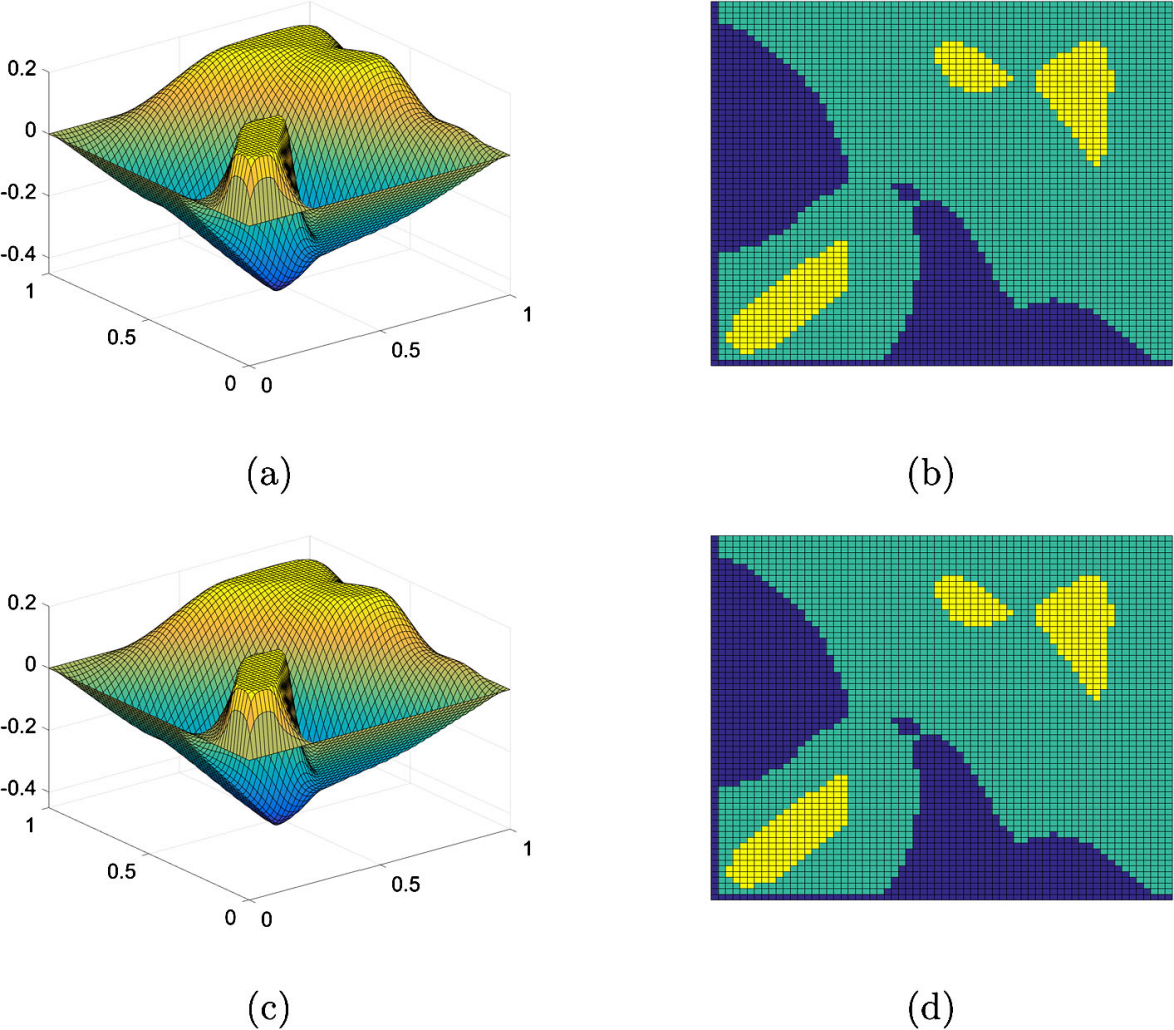
**The numerical solutions and the contact sets for the elastic-plastic torsion problem at the** $\pmb{64\times64}$ **resolution.** **(a)**-**(b)** by the PDL1P and **(c)**-**(d)** by the obstacle SOR.

In Table 3, we present performances of the PDL1P and the obstacle SOR applied for the elastic-plastic torsion problem (5.7). For the PDL1P, we use the parameters suggested in [14] ($\mu=0.1$, $r_{1}=0.008$, $r_{2}=15.625$). As one can see from the table, our suggested method again resulted in the numerical solution about one order faster than the PDL1P measured in the CPU time. In Figure 7, we illustrate the simulated membranes in the equilibrium satisfying (5.7) and their contact sets at resolution $64\times64$. In Figures 7(b) and 7(d), the upper and lower contact sets are colored in yellow (brightest in gray scale) and blue (darkest in gray scale), respectively. The results produced by the two methods are *apparently* the same.

**Table 3:** **The number of iterations and the CPU time for the elastic-plastic torsion problem (** **5.7** **)**

$\boldsymbol{(\varepsilon =10^{-6})}$	**PDL1P**		**Obstacle SOR**	
**Resolution**	**Iter**	**CPU**	**Iter**	**CPU**
32 × 32	887	0.13	47	0.02
64 × 64	1,287	0.68	98	0.04
128 × 128	1,609	3.43	193	0.22
256 × 256	1,866	17.27	368	1.58

For the PDL1P, we use the parameters suggested in [14] (*μ* = 0.1, $r_{1}=0.008$, $r_{2}=15.625$).

### Nonlinear obstacle problems

The obstacle SOR is implemented for nonlinear obstacle problems as described in Section 3.2.

**Figure 8:**
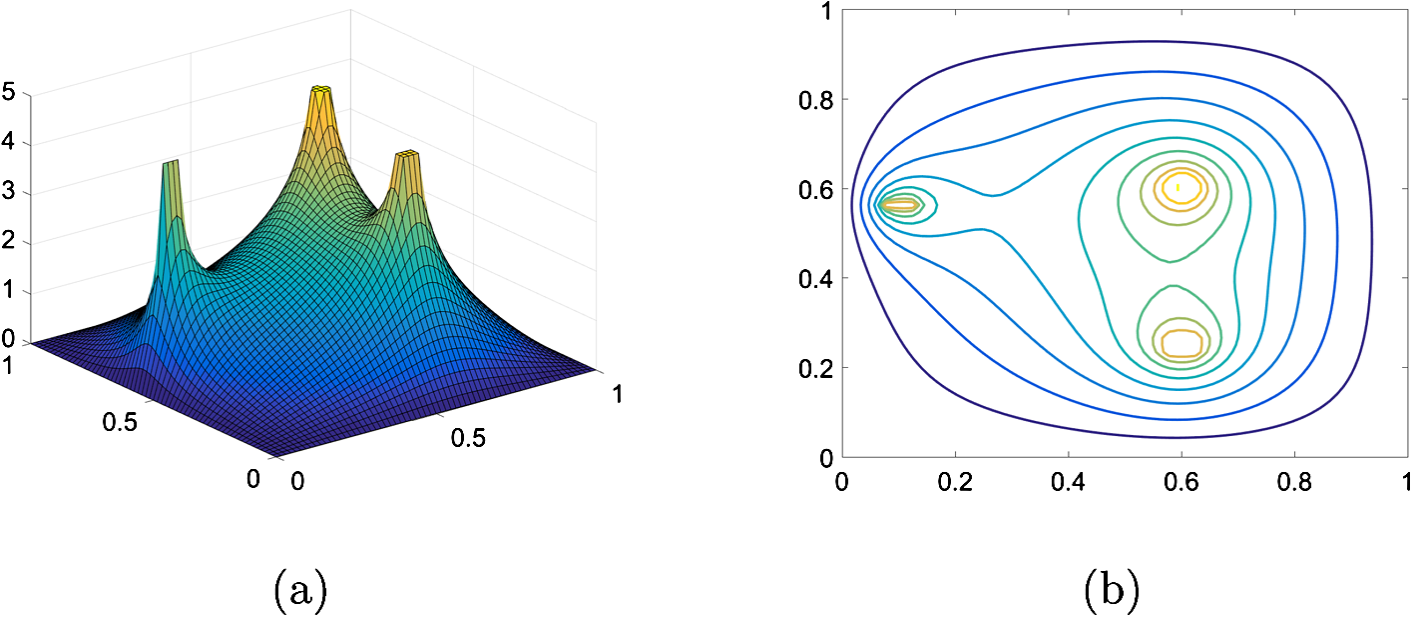
**Nonlinear obstacle problem with** $\pmb{\varphi =\varphi _{1}}$ **at resolution** $\pmb{64\times64}$ **.** **(a)** The nonlinear numerical solution and **(b)** its contour plot.

**Figure 9:**
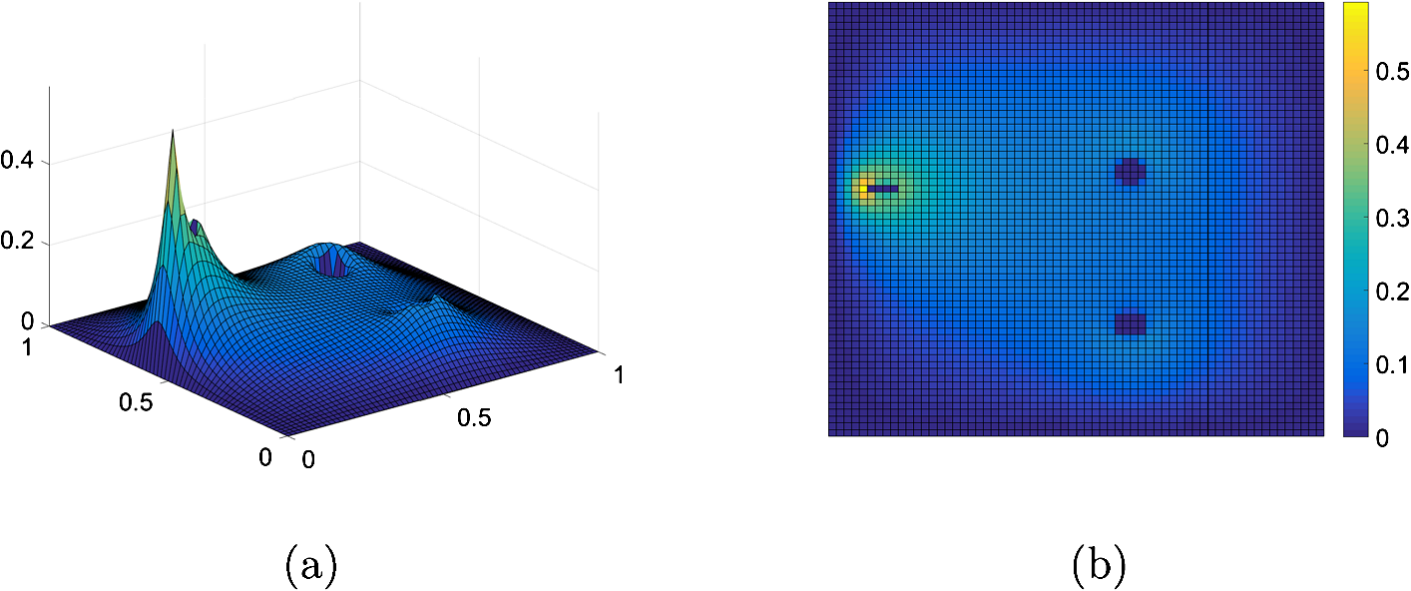
**The difference between the linear solution and the nonlinear solution, at the** $\pmb{64\times64}$ **resolution, for the obstacle problem with** $\pmb{\varphi =\varphi _{1}}$ **.** **(a)** $(u_{h,\text{linear}}-u_{h,\text{nonlinear}})$ and **(b)** its density plot.

In Table 4, we present experiments for which the obstacle SOR is applied for nonlinear obstacle problems with $\varphi =\varphi _{i}$, $i=1,2,3$. From a comparison with linear cases presented in Tables 1, 2, and 3, we can see for each of the obstacles that the obstacle SOR iteration for the nonlinear problem converges in a similar number of iterations as for the linear problem. Only the apparent difference is the CPU time; an iteration of the nonlinear solver is about as six time expensive as that of the linear solver, due to the computation of coefficients as in (3.19). For $\varphi =\varphi _{1}$, the nonlinear solution is plotted in Figure 8. Compared with the linear solutions in Figure 3, the nonlinear solution shows slightly lower function values, which is expected. As the grid point approaches the obstacles, the solution shows an increasing gradient magnitude. This may enlarge weights for far-away grid values as shown in (3.19), which in return acts as a force to reduce function values. The difference between the linear solution and the nonlinear solution, at the $64\times64$ resolution, is depicted in Figure 9.

**Table 4:** **The performance of the obstacle SOR applied for nonlinear obstacle problems with** $\pmb{\varphi =\varphi _{i}}$ **,** $\pmb{i=1,2,3}$

$\boldsymbol{(\varepsilon =10^{-6})}$	$\boldsymbol{\varphi _{1}}$		$\boldsymbol{\varphi _{2}}$		$\boldsymbol{\varphi _{3}}$	
**Resolution**	**Iter**	**CPU**	**Iter**	**CPU**	**Iter**	**CPU**
32 × 32	79	0.02	62	0.04	47	0.04
64 × 64	133	0.16	121	0.17	98	0.16
128 × 128	257	1.25	239	1.16	192	1.09
256 × 256	513	10.19	477	9.20	368	8.24

### Post-processing algorithm

In Figure 5, one have seen that the error, the difference between the numerical solution and the analytic solution, shows its highest values near the free boundary. The larger error is due to the result of mismatch between the mesh grid and the obstacle edges. In order to eliminate the error effectively, we apply the subgrid FD schemes in Section 4 as a post-processing (PP) algorithm. For the examples presented in this subsection, the numerical solutions are solved as follows: (a) the problem is solved with $\varepsilon =10^{-5}$ (pre-processing), (b) the free boundary is estimated with $(k_{0},k_{1})=(10,4)$ and subgrid FD schemes are applied at neighboring grid points as in Section 4, and (c) another round of iterations is applied to satisfy the tolerance $\varepsilon =10^{-7}$.

First, we consider a step function for an one-dimensional (1D) obstacle, as in Section 2.2. Let $\varOmega =[0,1]$ and $\varphi_{4}:\varOmega \rightarrow \mathbb{R}$ defined by

5.10 $$ \varphi _{4}(x)=\left \{ \textstyle\begin{array}{l@{\quad}l} 0 &\mbox{if }0\le x< \pi/6,\\ 1 &\mbox{if }\pi/6\le x\le1. \end{array}\displaystyle \right . $$

The analytic solution to the linear problem is given as

5.11 $$ u_{4,true}(x)=\left \{ \textstyle\begin{array}{l@{\quad}l} 6x/\pi&\mbox{if }0\le x< \pi/6,\\ 1 &\mbox{if }\pi/6\le x\le1. \end{array}\displaystyle \right . $$

**Figure 10:**
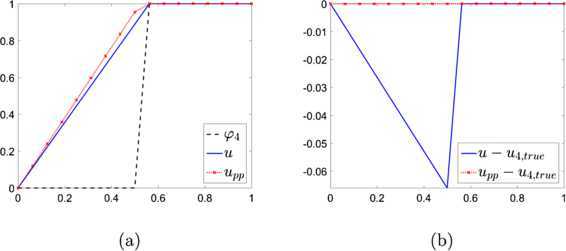
**The post-processing algorithm applied for an obstacle problem in 1D at resolution 16.** **(a)** The computed solutions without the post-process (blue solid curve) and with the post-process (red dotted curve with × marks) and **(b)** their errors. Here the subscript pp indicates the post-process.

Figure 10 shows the numerical solutions to the linear problem associated to (5.10) with and without the post-process, and their errors. The numerical solutions without and with the post-process are obtained iteratively satisfying the tolerance $\varepsilon =10^{-7}$. Notice that the solution without post-process is underestimated and shows a relatively high error: $\|u-u_{4,\mathrm{true}}\|_{\infty}=0.066$. The error is reduced to $\|u_{\mathrm{pp}}-u_{4,\mathrm{true}}\|_{\infty}=8.57\times 10^{-7}$ after the post-process.

The post-processing algorithm is applied to the linear obstacle problem in 2-D involving $\varphi =\varphi _{2}$. Table 5 contains efficiency results that compare performances of the PDL1P, the obstacle SOR (without post-process), and the obstacle SOR with the post-process (Obstacle SOR+PP) at various resolutions; while Table 6 presents an accuracy comparison for those methods. According to Table 5, the post-processed solution requires about 40% more iterations than the non-post-processed one; the incorporation of the post-process makes the iterative algorithm as twice expensive measured in CPU time as the original iteration. However, one can see from Table 6 that the post-process makes the error reduced by a factor of $15{\sim}20$. Thus in order to achieve a three-digit accuracy in the maximum-norm, for example, the PDL1P requires 10.47 seconds and the obstacle SOR completes the task in 0.84 seconds; when the obstacle SOR+PP takes only 0.1 second.

**Table 5:** **CPU time and iteration comparisons for the suggested post-process, applied to the linear problem with** $\pmb{\varphi =\varphi _{2}}$

$\boldsymbol{(\varepsilon =10^{-7})}$	**PDL1P**		**Obstacle SOR**		**Obstacle SOR+PP**	
**Resolution**	**Iter**	**CPU**	**Iter**	**CPU**	**Iter**	**CPU**
25 × 25	814	0.07	56	0.02	92	0.07
50 × 50	1,486	0.40	108	0.03	147	0.10
100 × 100	2,092	2.26	213	0.13	299	0.26
200 × 200	2482	10.47	416	0.84	570	1.34

**Table 6:** $\pmb{L^{\infty}}$ **and** $\pmb{L^{2}}$ **error comparisons for the suggested post-process, applied to the linear problem with** $\pmb{\varphi =\varphi _{2}}$

$\boldsymbol{(\varepsilon=10^{-7})}$	**PDL1P**		**Obstacle SOR**		**Obstacle SOR+PP**	
**Resolution**	$\boldsymbol{L^{\infty}}$ **error**	$\boldsymbol{L^{2}}$ **error**	$\boldsymbol{L^{\infty}}$ **error**	$\boldsymbol{L^{2}}$ **error**	$\boldsymbol{L^{\infty}}$ **error**	$\boldsymbol{L^{2}}$ **error**
25 × 25	1.94⋅10^−2^	4.35⋅10^−3^	1.94⋅10^−2^	4.38⋅10^−3^	8.44⋅10^−4^	2.80⋅10^−4^
50 × 50	4.38⋅10^−3^	8.10⋅10^−4^	4.39⋅10^−3^	8.41⋅10^−4^	2.01⋅10^−4^	6.93⋅10^−5^
100 × 100	1.25⋅10^−3^	2.73⋅10^−4^	1.25⋅10^−3^	2.87⋅10^−4^	5.16⋅10^−5^	1.79⋅10^−5^
200 × 200	5.45⋅10^−4^	7.29⋅10^−5^	5.46⋅10^−4^	7.76⋅10^−5^	1.40⋅10^−5^	4.74⋅10^−6^

**Figure 11:**
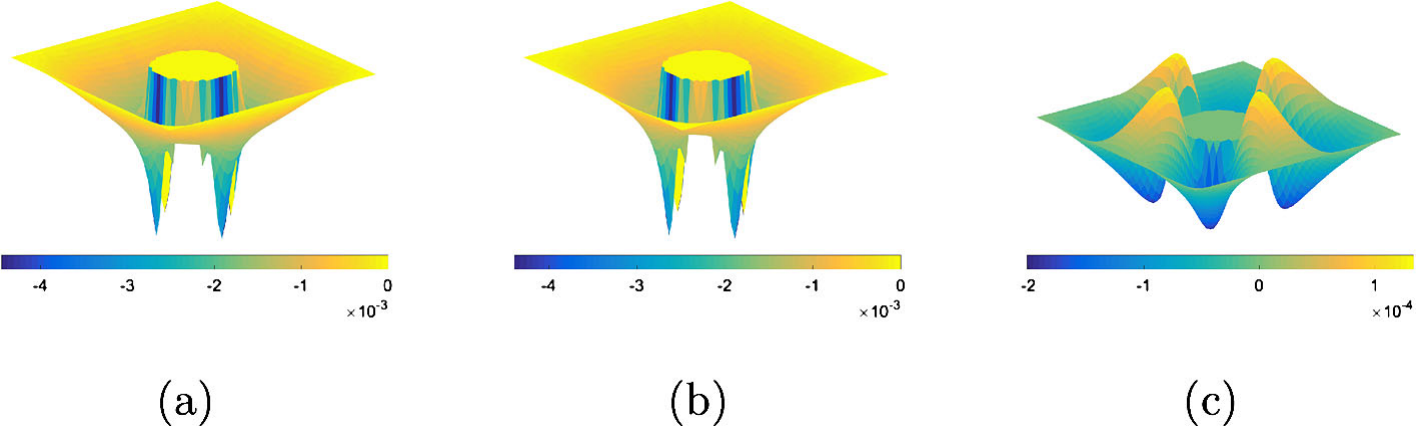
**Plots of the error (** $\pmb{u_{h}-u^{*}}$ **) at the** $\pmb{50\times 50}$ **resolution for the linear obstacle problem with** $\pmb{\varphi =\varphi _{2}}$ **.** **(a)** by the PDL1P, **(b)** by the obstacle SOR, and **(c)** by the obstacle SOR+PP.

Figure 11 includes plots of the error ($u_{h}-u^{*}$) at the $50\times50$ resolution for the linear obstacle problem with $\varphi =\varphi _{2}$, produced by the PDL1P, the obstacle SOR, and the obstacle SOR+PP. The numerical solutions of the PDL1P and the obstacle SOR are almost identical to each other and clearly underestimated, with the maximum discrepancy occurring around the free boundary due to the misfit between the mesh grid and the free boundary. It can be seen from Figure 11(c) that the post-process can eliminate the misfit error very effectively; the remaining error is the truncation error introduced by the second-order FD schemes.

### Parameter choices

Finally, we present experimental results for parameter choices, when the obstacle SOR is applied for the linear problem with $\varphi =\varphi _{3}$. For an effective calibration of the optimal relaxation parameter as suggested in (3.28), we first select $h_{0}=1/25$. Then by using a trial-by-error method, we found the calibrated optimal relaxation parameter $\widehat{\omega}_{h_{0}}=1.61$, which results in the following calibrated constant:

5.12 $$ c_{0}\approx6.0559. $$

Thus it follows from (3.27) that the calibrated optimal relaxation parameter reads

5.13 $$ \widehat{\omega}_{\mathrm {cal},h}\approx \left \{ \textstyle\begin{array}{l@{\quad}l} 1.6817 & \mbox{when }h=1/32,\\ 1.8371 & \mbox{when }h=1/64,\\ 1.9097 & \mbox{when }h=1/128,\\ 1.9538 & \mbox{when }h=1/256, \end{array}\displaystyle \right . $$

which is used for the results of the obstacle SOR included in Table 3.

**Figure 12:**
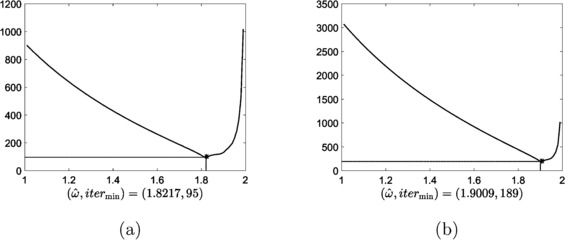
**The relaxation parameter** ***ω*** **(horizontal axis) vs. the number of iterations (vertical axis) for solving the linear obstacle problem with** $\pmb{\varphi =\varphi _{3}}$ **by the obstacle SOR.** **(a)** when $h=1/64$ and **(b)** when $h=1/128$.

In order to verify effectiveness of the calibration, we implement a line search algorithm to find a relaxation parameter *ω̂* that converges in the smallest number of iterations with $\varepsilon =10^{-6}$, the same tolerance as for the results in Table 3. For $h=1/64$ and $h=1/128$, the line search algorithm returned the curves as shown in Figure 12 with

5.14 $$ [\widehat{\omega},\mbox{iter}] \approx \left \{ \textstyle\begin{array}{l@{\quad}l} [1.6732, 45 ] & \mbox{when }h=1/32,\\ {[1.8217, 95 ]} & \mbox{when }h=1/64,\\ {[1.9009, 189 ]} & \mbox{when }h=1/128. \end{array}\displaystyle \right . $$

Note that when the calibrated parameters are used, the iteration counts of the obstacle SOR presented in Table 3 are 47, 98, and 193, respectively, for $h=1/32$, $h=1/64$, and $h=1/128$. Thus the calibrated optimal parameters in (5.13) are quite accurate for the optimal convergence.

## Conclusions

Although various numerical algorithms have been suggested for solving elliptic obstacle problems effectively, most of the algorithms presented in the literature are yet to be improved for both accuracy and efficiency. In this article, the authors have studied obstacle relaxation methods in order to get second-order finite difference (FD) solutions of obstacle problems more accurately and more efficiently. The suggested iterative algorithm is based on one of the simplest relaxation methods, the successive over-relaxation (SOR). The iterative algorithm is incorporated with subgrid FD methods to reduce accuracy deterioration occurring near the free boundary when the mesh grid does not match with the free boundary. For nonlinear obstacle problems, a method of gradient-weighting has been introduced to solve the problem more conveniently and efficiently. The iterative algorithm has been analyzed for convergence for both linear and nonlinear obstacle problems. An effective strategy is also presented to find the optimal relaxation parameter. The resulting obstacle SOR has converged about one order faster than state-of-the-art methods and the subgrid FD methods could reduce the numerical errors by one order of magnitude.
